# Knowledge mapping and visualization of trends in immunotherapy for ovarian cancer over the past five years: a bibliometric analysis

**DOI:** 10.3389/fimmu.2024.1465917

**Published:** 2024-10-03

**Authors:** Kowthar Mohamed Shaie, Lian Sihan, Wang Yuli, Han Mengfei, Feng Renqian, Hu Yan

**Affiliations:** Department of Gynecology, First Affiliated Hospital of Wenzhou Medical University, Wenzhou, Zhejiang, China

**Keywords:** immunotherapy, ovarian cancer, knowledge mapping, scientific trends, bibliometric analysis, Web of Science

## Abstract

**Background:**

This study conducts a bibliometric literature analysis to explore trends in immunotherapy for ovarian cancer from 2019 to 2023.

**Methods:**

An extensive online literature search was conducted in the Web of Science Core Collection database to identify English-language articles and reviews related to “trends in immunotherapy”, and “ovarian cancer”. statistical analysis was performed using VOSviewer to visualize and compare nations, institutions, and journals simultaneously.

**Results:**

Our findings highlight contributions by 118 nations, led by the People’s Republic of China with 3,167 contributions; Germany followed with 558 and Italy having 547. Of all publications made between 2019-2023, “Frontiers Immunology” had the most publications with 546 total records followed by “cancers “, and “frontiers in oncology” being the most heavily relied upon categories. Annually publication trends increased until 2022 but then declined considerably as a peak of highly-cited papers occurring between 2019 and 2022.

**Conclusions:**

Our bibliometric analysis not only maps the evolution of immunotherapy research in ovarian cancer but also provides actionable insights for advancing scientific progress. By identifying emerging trends and key areas, future research can strategically enhance treatment strategies and outcomes for ovarian cancer patients.

## Introduction

Ovarian cancer is a significant health concern for women worldwide. Current estimates indicate that in 2020 each year over 300,000 new instances of ovarian cancer were identified, making up 3.4% of all newly diagnosed cancer cases and contributing to 4.7% of cancer-related deaths among women (1). In 2022, there were about twenty thousand new cases of ovarian cancer reported in the United States, leading to approximately twelve thousand eight hundred and ten deaths among women from the disease (2). Thereby most several risk factors associated with ovarian cancer, including excessive weight, breast cancer in the family, smoking, early or late menstruation, and reproductive issues (3). Due to nonspecific manifestations, over 70% of ovarian cancer cases are often detected at a late stage (4). In the majority of countries, the likelihood of survival rate for ovarian cancer is typically below 40 out of 100 (5). The lack of early and effective detection methods is the primary reason for the dismal prognosis and elevated mortality rate (6) The first-line and standard treatments for ovarian cancer (OC) include tumor removal surgery and platinum-based chemotherapy. Tumor removal surgery aims to ensure the maximum resection of all visible tumors and accurate tumor staging, with optimal debulking typically defined as having residual tumors less than 1 cm in diameter per nodule (7). Therefore, it is necessary to increase efforts to identify and understand novel immunotherapies and specific targeted therapies for ovarian cancer, as well as to develop more effective diagnostic and treatment strategies.

In the past decades, the application of immunotherapy has significantly expanded the scope of treatment methods, demonstrating favorable prognostic impacts across various types of cancers (8–10). However Ovarian cancer (OC) exhibits limited response to immunotherapy (11). Genome sequencing over the past decade has progressively revealed insights into OC (12). Identifying driver mutations in ovarian cancer (OC) using initiatives like the Cancer Genome Atlas (TGGA) provides novel therapeutic strategies (12). The predictive ability of immunotherapy outcomes can be improved by evaluating sensitive and resistant targeted therapy subgroups represented by biomarkers (11, 13). Further immunotherapy Treatments, such as adoptive T-cell therapy and vaccines, are currently under investigation (14). Bibliometrics is a key tool for evaluating scientific and technological progress, aiding in the exploration of both qualitative and quantitative research methods. Through systematic investigation, it clarifies data characteristics within the designated research field and depicts research content and trends via knowledge graphs (15–17). This method involves selecting articles within a specific research field and analyzing various aspects, such as the country, author, keyword, publishing agency, and journals by utilizing VOSviewer (18). Our study aimed to explore knowledge mapping and trends visualization in the field of immunotherapy for ovarian cancer over the past five years. Using Web of Science database, we investigated document systems and measurement methodologies as a research area, employing quantitative research methods to analyze the distribution, relationships, and changes across various domains.

## Materials and methods

### Data source

**Figure 1:**
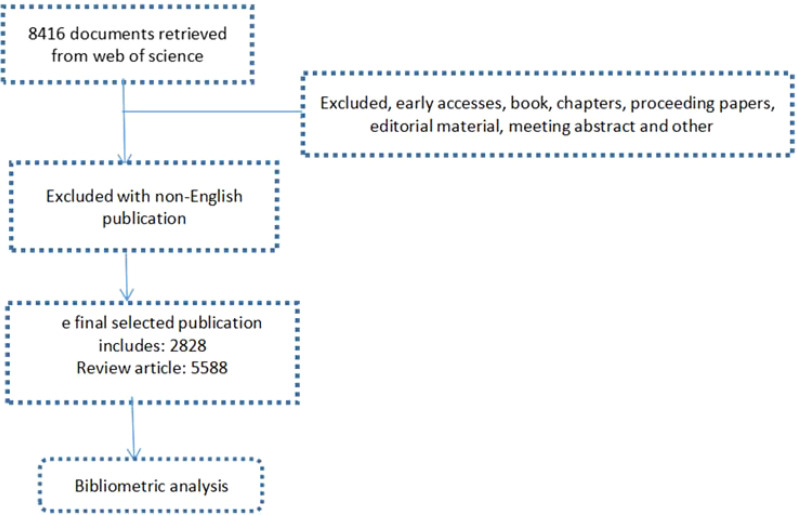
Displays the flowchart for analyzing research data.

This study utilizes data from the Core Collection of Web of science), focusing specifically on studies, including both articles and reviews, published between January 1, 2019, and December 31, 2023, exclusively in English. The formula of searching: TS = [“ovarian cancer” OR “ovarian tumor” OR “ovarian neoplasm” OR “malignant neoplasm of ovarian”] AND “immunotherapy” OR “immunotherapeutic” OR “immunotherapeutic response” OR “immunotherapeutic target”]. the specific process outlined in the flowchart is shown in **Figure 1**.

The search terms employed in this study were meticulously selected to formulate a comprehensive search strategy aimed at encompassing a broad spectrum of literature relevant to trends in immunotherapy for ovarian cancer.

### Data analysis

We downloaded data in TXT format from the Web of Science, and used VOSViewer version 1.6.20, as a software application that generates maps of scholarly works. VOSviewer focuses on a graphical representation to illustrate bibliometric relationships within its user interface (18). This tool facilitated the creation of network visualization maps for analyzing co-authorship, co-occurrence, and co-cited entities, including countries/regions, institutions, authors, and keywords of interest. In these visualization maps, node size corresponds to the frequency of relevant elements, chain thickness indicates the strength of connections, and node color represents affiliation with distinct modules. This approach enables a comprehensive examination of collaborative relationships, thematic intersections, and influential entities within the scholarly landscape, thereby enhancing our understanding of research network dynamics on a broader scale.

### Data mapping

VOS viewer software was employed for the generation of visualizations depicting the results of co-occurrence data analysis. The presented visual representation takes the form of a map, showcasing each keyword in distinct colors. The size of individual keyword clusters on the map correlates directly with their frequency of appearance in the article. Additionally, the spatial closeness of two keywords on the visualization map functions as an indicative measure of their co-occurrence. This method of visualization provides a nuanced understanding of the interrelationships between keywords, facilitating the identification of thematic concentrations and patterns emerging from the analyzed data.

### Bibliometric assessment

This article offers a thorough review and assessment of the research topic, conducting in-depth analyses of publishing models, institutional influences, country trends, keyword usage, and author patterns. The evaluation explores the intricacies of academic contributions, institutional impacts, and regional disparities, unveiling essential dimensions that mold the research landscape within specific fields. The adoption of bibliometric methods serves as a robust framework for systematically gauging academic impact and discerning significant trends. This approach contributes to a detailed understanding of research prospects associated with the examined topic, providing valuable insights for the scholarly community and practitioners alike.

## Results

### Annual publication trends

**Figure 2:**
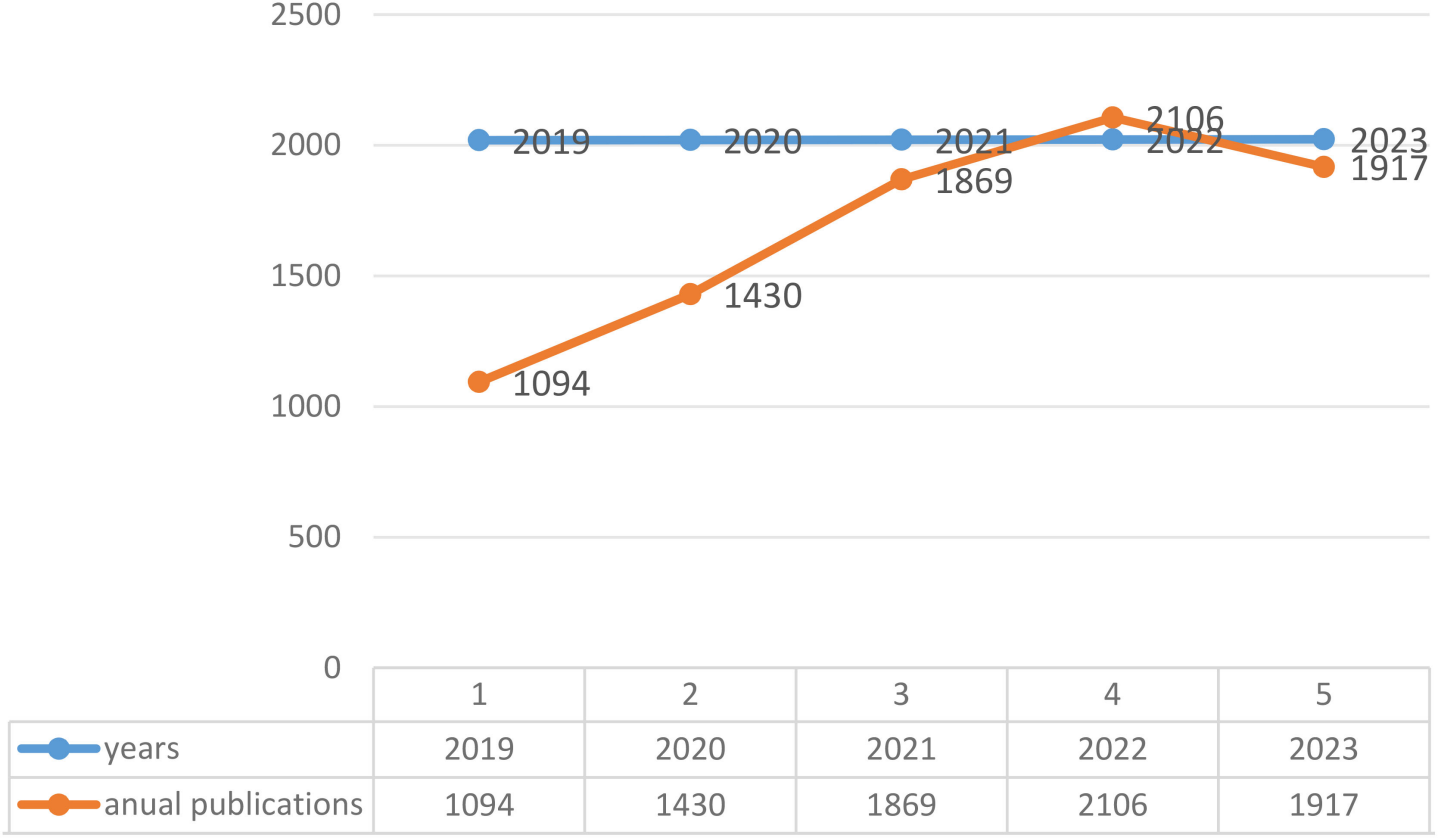
Annual publication representation of the temporal trends in the scholarly contributions to the field.

In our study, 8416 articles were incorporated, encompassing 5,588 research articles and 2,828 reviews with a focus on immunotherapy and ovarian cancer. The volume of publications related to immunotherapy and ovarian cancer exhibited an upward trajectory from 2019 to 2022. Notably, the data reveals a downturn in publications for the year 2023. Within the year 2022, a pinnacle was reached with a publication count of 2,106 articles, marking it as the year with the highest scholarly output. However, in 2023, a noticeable decline in the number of publications became apparent. A comprehensive depiction of this information is elucidated in **Figure 2**, providing a visual representation of the temporal trends in the scholarly contributions to the field.

### Author insight analysis

**Figure 3:**
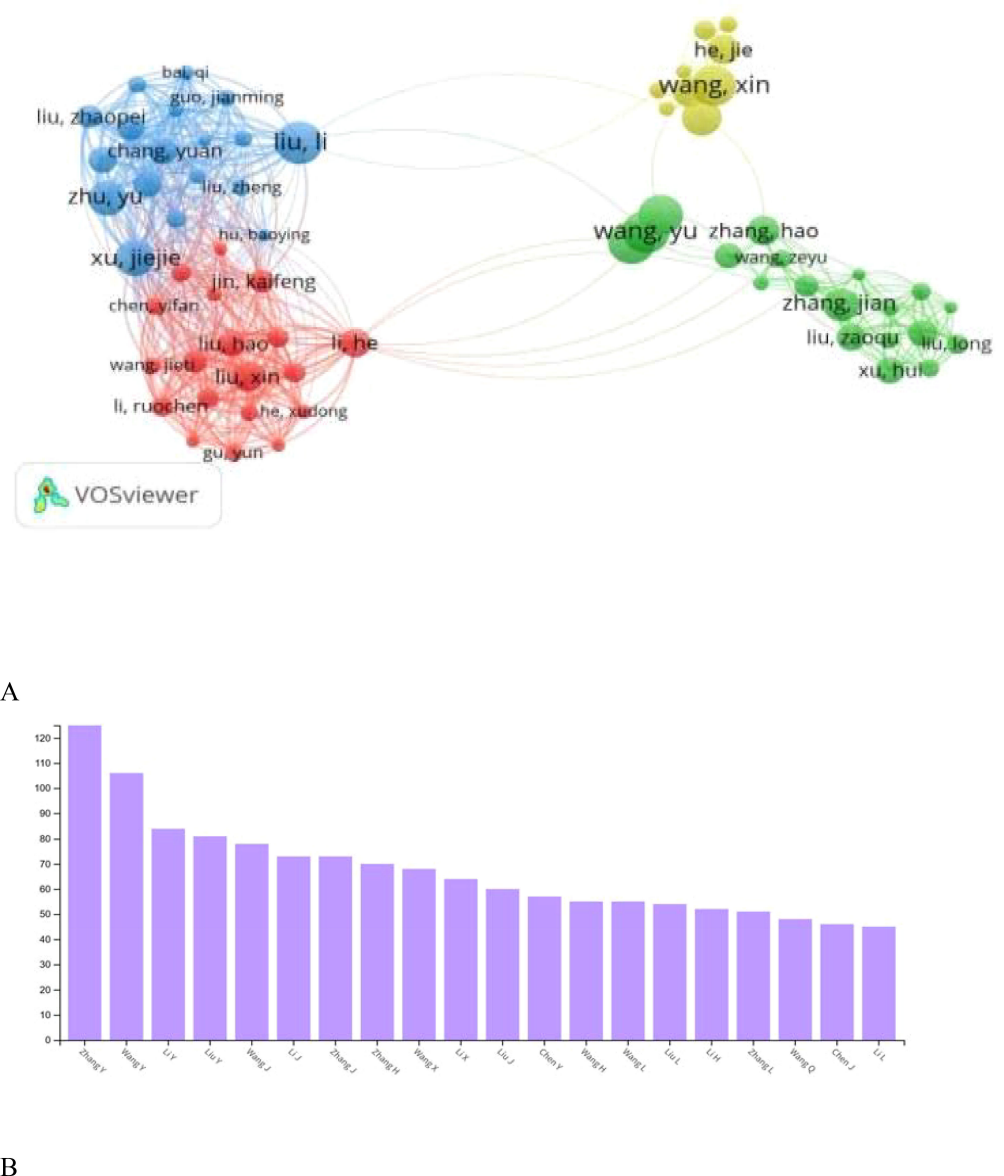
**(A)** Shows cross-citing relationships, and network visualization among Authors, while **(B)** shows the publishing productivity of different authors expressed as the number of publications.

Throughout the period from 2019 to 2023, a total of 48,238 authors have actively contributed to publications within the designated topic. **Figure 3B** delineates the top 20 authors, ranked according to their prolificacy in the realm of immunotherapy and ovarian carcinoma research. Notably, Zhang Y emerges as a frontrunner, boasting an impressive 125 published documents, exemplifying a substantial impact on the field. Close behind is Wang Y with 106 publications, securing the second position, while LiY occupies the third position with 84 publications. To provide a visual representation of the collaborative ties among these leading authors, **Figure 3A** illustrates the co-authorship connections within the top 20 authors in this comprehensive study.

### Analyzing contributions of different regions/countries

**Figure 4:**
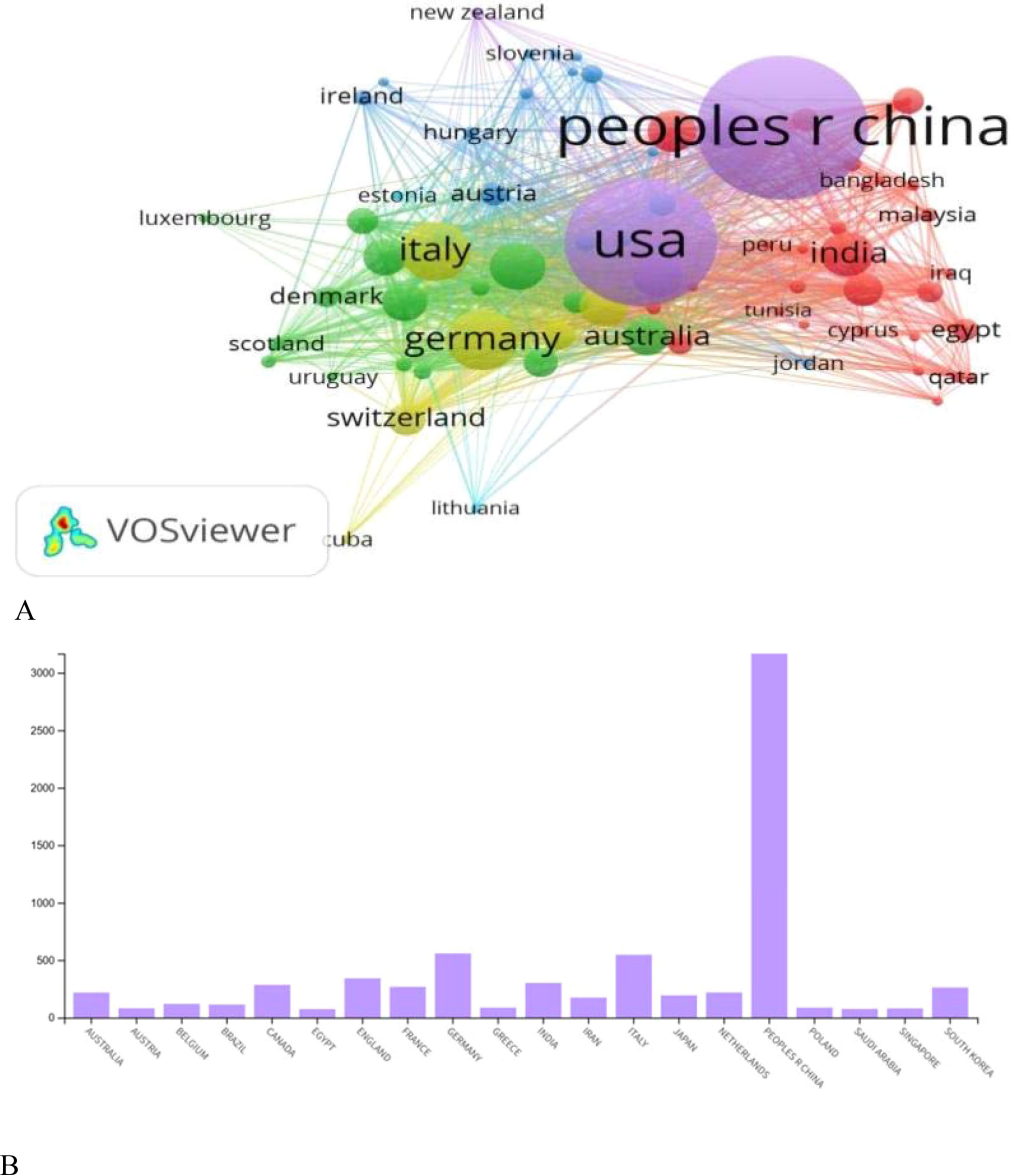
**(A)** Illustrates a network visualization map of the co-authorship networks among the countries, while **(B)** shows the most published regions and their publication numbers.

Over the last five years,118 nations have Provided contributions to the publication of papers in the designated Domain. **Figure 4B** provides an overview of the top 20 countries, systematically ranked based on their publication counts. The People’s Republic of China secures the foremost position with an impressive 3,167 publications, demonstrating a substantial research output. Following closely is Germany, contributing significantly with 558 publications, and Italy claiming the third position with 547 publications. Other notable contributors include England, Canada, and France, each presenting a substantial number of publications with 342, 284, and 268 documents, respectively. Additionally, South Korea, Netherlands, Spain, and Japan stand out among the top 20 countries in terms of scholarly contributions. To visually elucidate the collaborative connections between countries, **Figure 4A** offers a comprehensive representation of co-authorship networks within this global research landscape.

### Institutional contributions analysis in the publication

**Figure 5:**
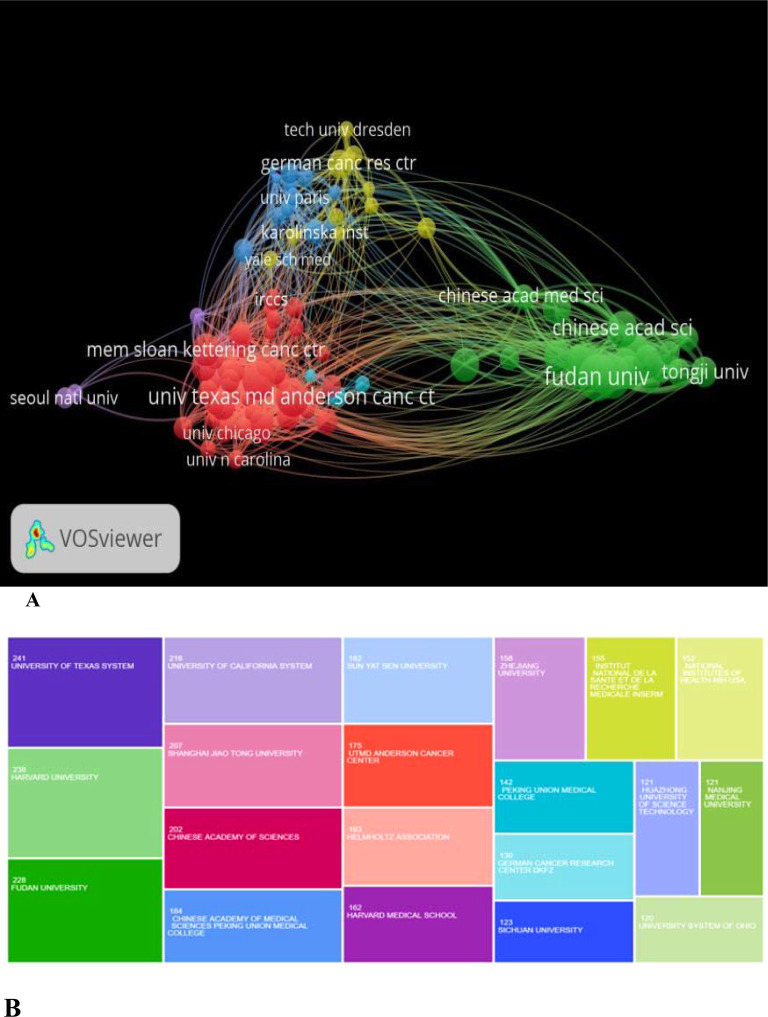
**(A)** Illustrates the co-authorship networks among these institutions, while **(B)** shows the most highly active institutions in the field and their number of publications per institute.

The institution boasting the highest volume of publications is the University of Taxes, having authored 241 articles, constituting a substantial proportion of the overall publications. The People’s Republic of China occupies 10 positions within the top 20 institutions, contributing 1,668 articles. Following closely in the second and third positions are the United States of America and Germany, respectively. **Figure 5A** visually articulates the correlation between the timeline and the volume of articles produced. The top 5 institutions, contributing significantly to the scholarly output, include University of Texas, Harvard University, Fudan University, University of California, and Shanghai Jiao Tong University. **Figure 5B** shows the top 20 most highly active institutions in the field, further emphasizing the influential contributions of these institutions.

### Analytic keywords

**Figure 6:**
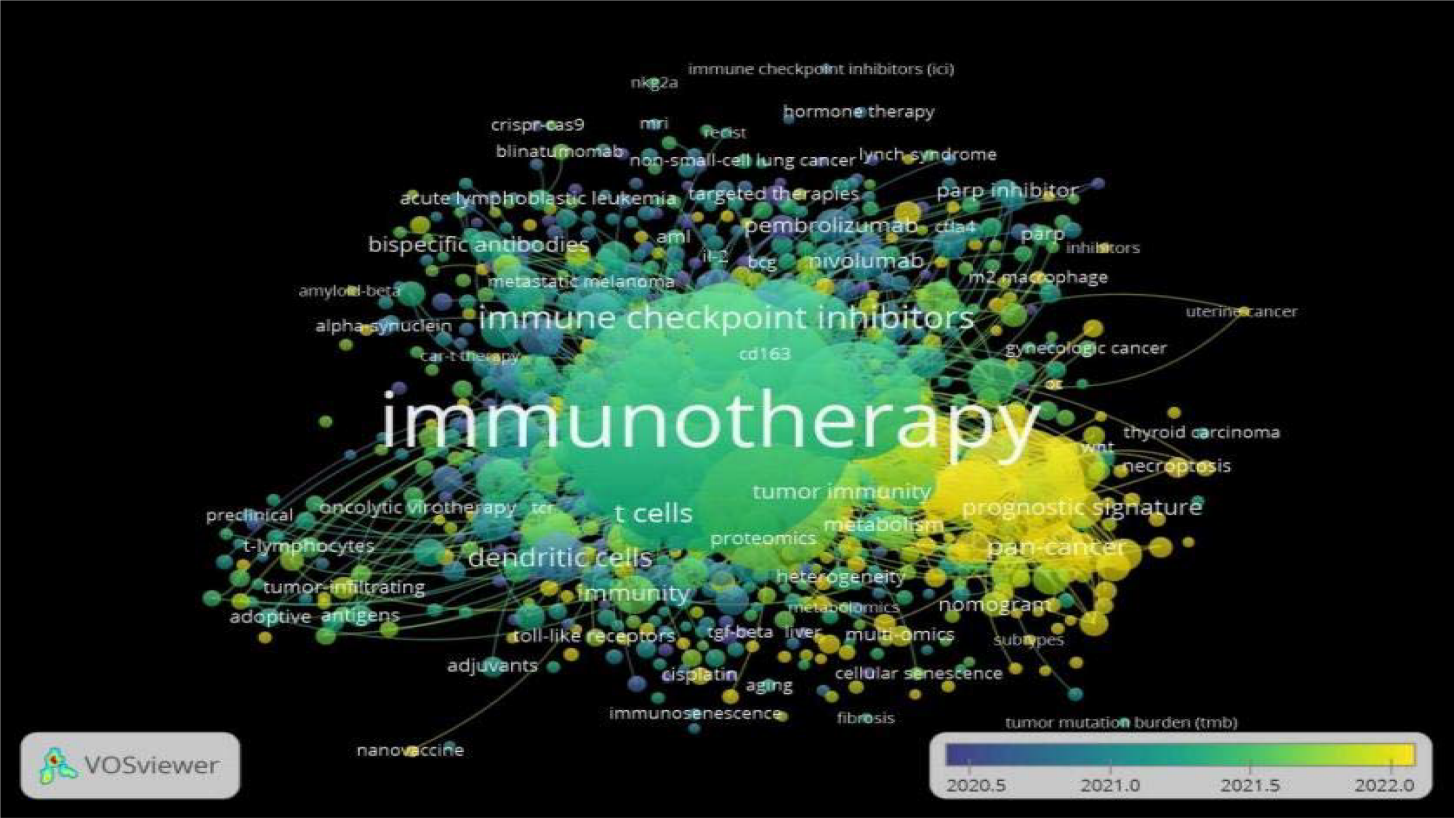
Demonstrates the visualization of the keyword co-occurrence coverage.

A total of 12,732 keywords are presented in **Figure 6**. The keywords were categorized into four distinct and color-coded clusters following keyword clustering analysis: blue, purple, green, and yellow. The dark blue cluster primarily encompasses terms like ‘immune checkpoint inhibitors’, ‘CRISPR-Cas9’, and ‘blinatumomab’. These terms were frequently used around mid-2020. the light blue cluster, keywords include terms like ‘t cells’, ‘dendritic cells’, and ‘tumor-infiltrating’. These terms were prevalent from early 2021 to mid-2021. The purple cluster includes terms like ‘PARP inhibitors, ‘hormone therapy’, and ‘targeted therapies. These terms used slightly more recently than the dark blue cluster, around late 2020 to early 2021. The green cluster keyword includes terms like ‘immunotherapy’, ‘adjuvants’, and ‘Nano vaccine’. These terms are commonly used around mid-2021 to early 2022. While the yellow cluster keywords include terms like ‘pan-cancer’, ‘prognostic signature’, and ‘necroptosis’. These terms indicate that have become prominent more recently, closer to 2022. This analytical approach provides a comprehensive understanding of key themes and concepts in the trend of immunotherapy in ovarian cancer. The visually represented clusters aid in identifying cohesive groups of keywords, assisting researchers in navigating and comprehending the multifaceted landscape of this scholarly domain. **Table 1** highlights the top 20 keywords in cancer immunotherapy research.

**Table 1:** Highlights the top 20 keywords in cancer immunotherapy research.

Ranking	Top 20 keyword	frequency	total link strength
1	cancer immunotherapy	3394	5730
2	ovarian-cancer	1608	3056
3	tumor-infiltrating lymphocytes	1381	2563
4	regulatory t-cells	876	1756
5	pd-l1	844	1827
6	open-label	728	1398
7	activation	681	1209
8	blockade	643	1488
9	dendritic cells	637	1185
10	cells	628	1352
11	ovarian cancer	587	1260
12	chemotherapy	581	1234
13	prognosis	538	933
14	therapy	478	867
15	survival	422	999
16	T-cells	401	720
17	Tumor-micro environment	358	570
18	cancer	338	617
19	expression	319	776
20	immunotherapy	288	676

### Contribution of journals

**Figure 7:**
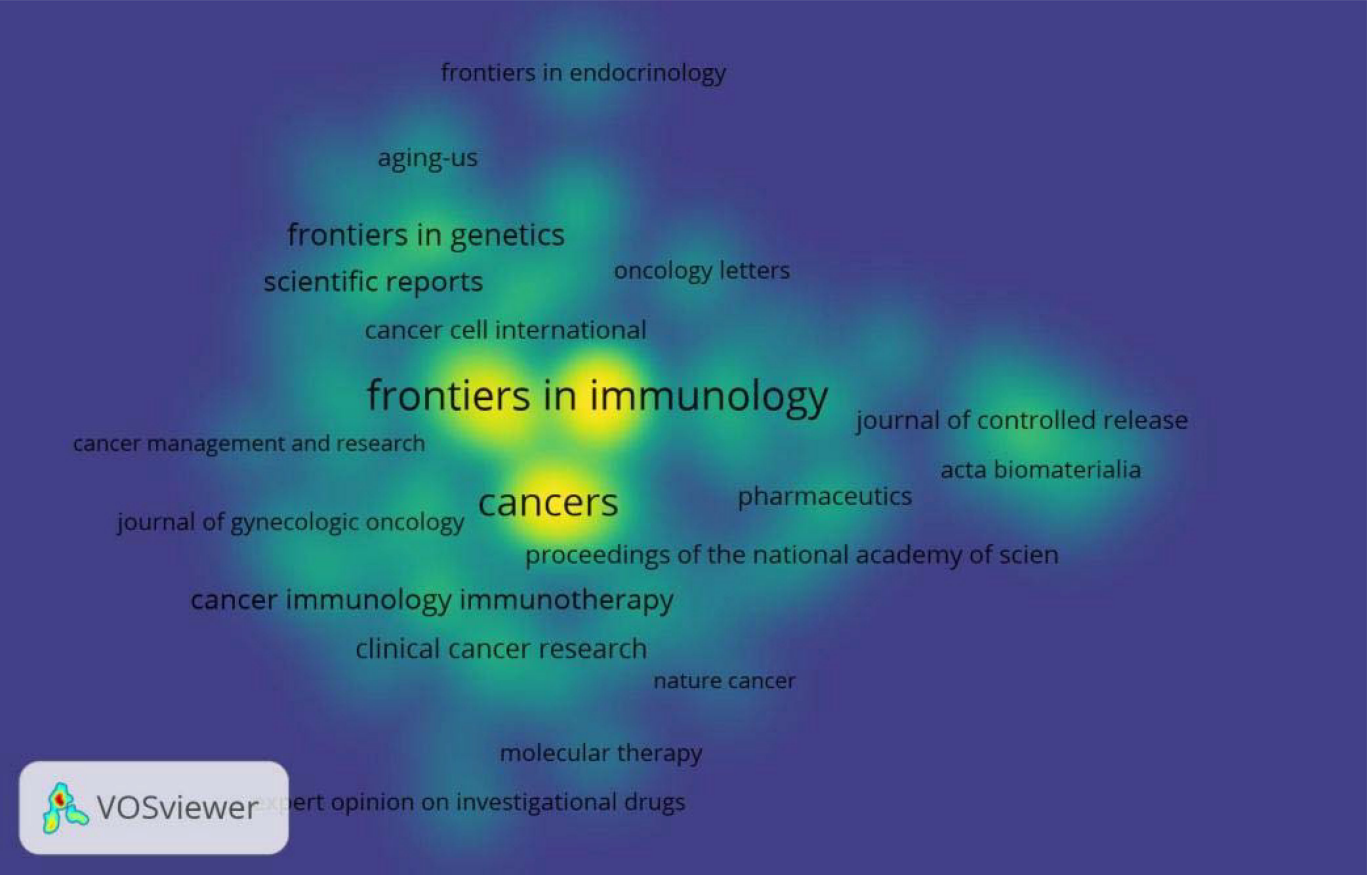
Presents a visual map illustrating the contribution of journals in 2019 to 2023.

It is estimated that 15,09 journals have disseminated Documents associated with this topic, with **Table 2** highlighting the top 20 most productive journals for immunotherapy and ovarian cancer research. Leading the list is the Journal of Frontiers Immunology, contributing a noteworthy 546 publications (IF=5.7, 6.40%). Following closely is the Cancers Journal (IF=4.5, 5.5%) and Frontiers in Oncology (IF=3.5, 3.2%) with 468 and 270 publications, respectively. International journal of Molecular Sciences ranks as the fourth most published journal with 217 publications, succeeded by the Journal for Immunotherapy of Cancer with 194 publications. The Nature Reviews Clinical Oncology Journal boasts the top-related impact factor (IF: 81.1), afterward annals of oncology (IF=56.7). The United States of America claims 8 positions within the top 20 most co-cited journals in this field. **Figure 7** provides a visualization map illustrating the contribution of journals, offering a dynamic representation of their interconnection and impact within the scholarly landscape.

**Table 2:** Highlights the top 20 most productive journals in cancer immunotherapy research.

Ranking	Co-cited journals	Records	Percentage%	IF 2023	countries
1	frontiers in immunology	546	6.40%	5.7	Switzerland
2	cancers	468	5.50%	4.5	Switzerland
3	frontiers in oncology	270	3.20%	3.5	Switzerland
4	international journal of molecular sciences	217	2.50%	4.9	united states
5	journal for immunotherapy of cancer	194	2.30%	10.3	England
6	frontiers in genetics	116	1.30%	2.8	Switzerland
7	cancer immunology immunotherapy	91	1%	4.6	united states
8	oncoimmunology	89	1%	6.5	united states
9	cells	76	0.90%	5.1	Switzerland
10	frontiers in cell and developmental biology	65	0.70%	4.6	Switzerland
11	nature communications	60	0.70%	14.7	England
12	clinical cancer research	58	0.60%	10	united states
13	gynecologic oncology	41	0.40%	4.5	united states
14	advanced science	38	0.40%	14.3	united states
15	cancer immunology research	37	0.43%	8.1	united states
16	seminars in cancer biology	25	0.29%	12.1	England
17	nature	16	0.19%	50.5	England
18	nature reviews clinical oncology	13	0.15%	81.1	England
19	annals of oncology	9	0.10%	56.7	England
20	journal of clinical oncology	9	0.10%	42.1	united states

### Document analysis

In the past five years, 8,416 documents and papers have been Published in the field of immunotherapy for ovarian carcinoma. **Table 3** presents 20 of the most extensively cited documents, highlighting the impactful contributions within the scholarly landscape. The document titled “CAR-T cell therapy: current limitations and potential strategies” holds the leading position, published in the Blood Cancer Journal in 2021 with an impact factor of 12.9 and accumulating 889 citations. This groundbreaking study emphasizes immunotherapy’s significance as a crucial cancer treatment. Following closely is the second most cited document, “Epithelial ovarian cancer: Evolution of management in the era of precision medicine” published in Cancer Journal for Clinicians New in 2019, boasting an impact factor of 503.1 and garnered 841 citations. This document presents a comprehensive overview of advancements in managing epithelial ovarian cancer, particularly in precision medicine.

**Table 3:** Presents a list of the 20 most widely cited documents in the field of cancer immunotherapy research from 2019 -2023.

Ranking	Title name	Citations	Year of publication
1	CAR-T cell therapy: current limitations and potential strategies	889	2021
2	Epithelial ovarian cancer: Evolution of management in the era of precision medicine	841	2019
3	*In situ* sprayed bioresponsive immunotherapeutic gel for post-surgical cancer treatment	694	2019
4	CD24 signaling through macrophage Siglec-10 is a target for cancer immunotherapy	688	2019
5	Nanoparticle-Mediated Immunogenic Cell Death Enables and Potentiates Cancer Immunotherapy	649	2019
6	Activatable Polymer Nanoenzymes for Photodynamic Immunometabolic Cancer Therapy	629	2019
7	Advances in immunotherapy for hepatocellular carcinoma	624	2021
8	Immune and genomic correlates of response to anti-PD-1 immunotherapy in glioblastoma	531	2019
9	Cooperation between Constitutive and Inducible Chemokines Enables T Cell Engraftment and Immune Attack in Solid Tumors	440	2019
10	mRNA vaccine for cancer immunotherapy	431	2021
11	Antitumor activity and safety of pembrolizumab in patients with advanced recurrent ovarian cancer: results from the phase II KEYNOTE-100 study	415	2019
12	Improving cancer immunotherapy using nanomedicines: progress, opportunities and challenges	401	2020
13	Treatment of epithelial ovarian cancer	396	2019
14	The Society for Immunotherapy of Cancer consensus statement on immunotherapy for the treatment of squamous cell carcinoma of the head and neck (HNSCC)	388	2019
15	The application of nanoparticles in cancer immunotherapy: Targeting tumor microenvironment	382	2021
16	IOBR: Multi-Omics Immuno-Oncology Biological Research to Decode Tumor Microenvironment and Signatures	371	2021
17	B cells, plasma cells and antibody repertoires in the tumor microenvironment	350	2020
18	CD8+ T cell differentiation and dysfunction in cancer	340	2022
19	Targeting tumor-associated macrophages to synergize tumor immunotherapy	330	2021
20	Macrophage-Based Approaches for Cancer Immunotherapy	329	2021

**Figure 8:**
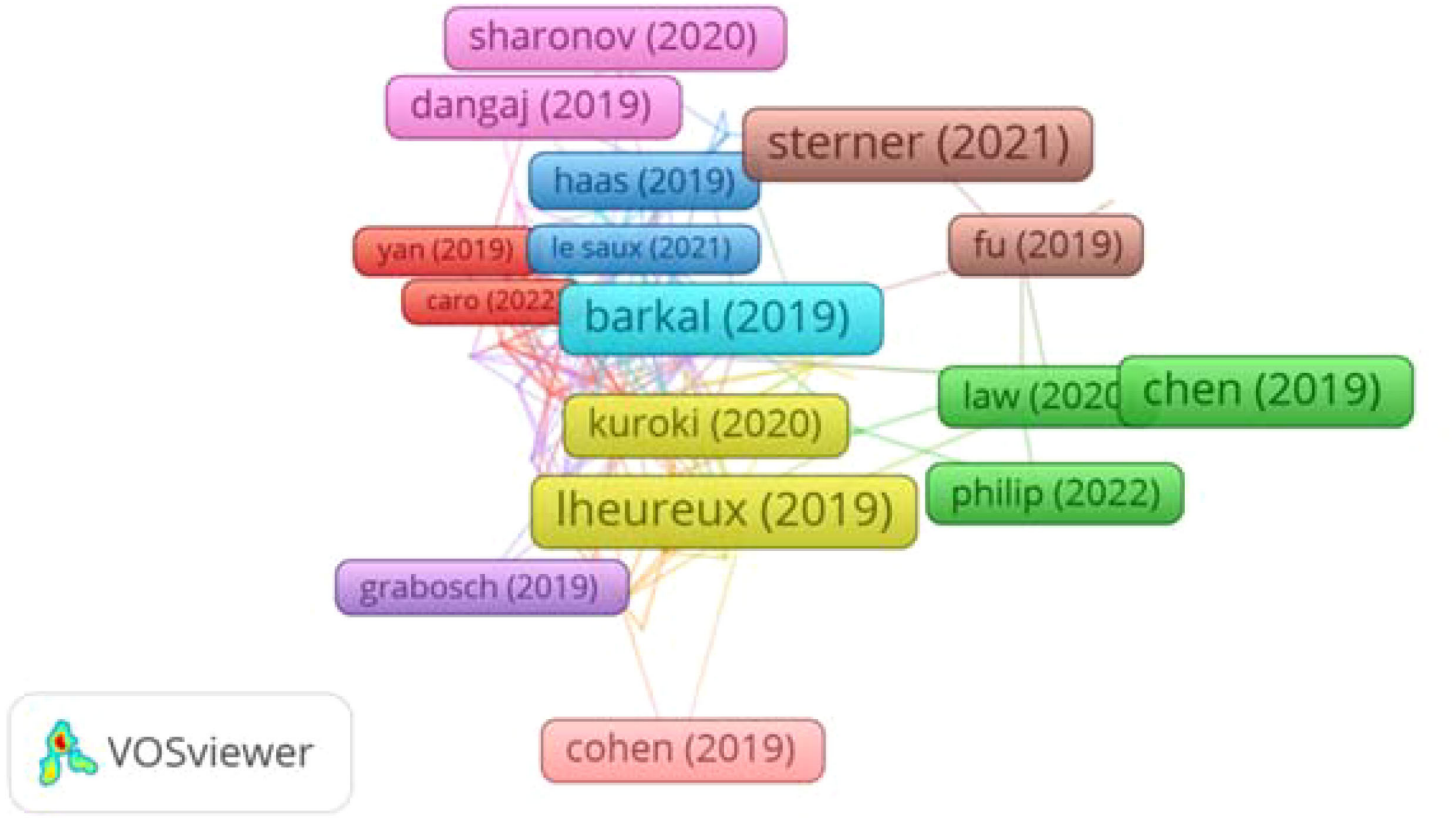
Illustrates a knowledge map related to authors of the widely cited documents.

Ranked third is the document titled “*In situ* sprayed bio responsive immunotherapeutic gel for post-surgical cancer treatment,” published in Nature Nanotechnology in 2019, holding an impact factor of 38.1, it has accumulated 694 citations. This report explores innovative post-surgical treatments using bio-responsive immunotherapeutic gels, offering promising strategies for improving patient outcomes. **Figure 8** visually presents knowledge maps of these widely referenced documents, offering a comprehensive overview of their interconnected themes and contributions. This visualization aids in understanding the key areas of focus and the relationships between influential studies in the field.

## Discussion

Immunotherapy has revolutionized tumor treatment by targeting key immune evasion mechanisms and stimulating CD8+ T cells to eliminate tumor cells. Across various cancers, like melanoma, colorectal cancer, and lung cancer, immune checkpoint inhibitors including anti-PD-1/PD-L1, anti-CTLA-4, and anti-Tim3, has significantly enhanced progression-free and overall survival rates. This success underscores their potential for ovarian cancer treatment, given its immunogenic nature. Biomarkers such as Regulatory T cells (Tregs), tumor-infiltrating lymphocytes (TILs), natural killer cells, and tumor-associated macrophages (TAMs) have been identified in blood, ovarian cancer tissue, and abdominal fluid, highlighting their role in immune response (19–25).

Nivolumab is the first clinically validated immune checkpoint inhibitor (ICI) for advanced cancer, and subsequent trials have further established the effectiveness of ICIs, in treating advanced and recurrent ovarian cancer (26–30). However, ICIs have demonstrated limited effectiveness in newly diagnosed ovarian cancer due to compensatory immune checkpoint mechanisms that restrict monotherapy efficacy (31, 32).

Our research provides unique insights and perspectives that contribute novel ideas to the field of immunotherapy and ovarian cancer treatment research. By conducting an exhaustive bibliometric analysis of trends of immunotherapy in OC literature published over the past five years, we offer an unparalleled view of research in this domain. This evaluation represents the first-ever ovarian cancer immunotherapy in bibliometrics; thus, demonstrating its groundbreaking significance within its field. By reviewing 8416 articles and reviews, Publications can have a profound effect on the productivity and progress of an area of study. We provide an in-depth view of research trends, prominent authors, and potential research gaps. The knowledge mapping and visualization aspects of our analysis reveal several important trends and transformations in the research field. For instance, there is a notable increase in focus on combination therapy, the utilization of biomarkers for treatment response prediction, and the geographical distribution of research activities. These visualizations not only aid in comprehending the current research landscape but also highlight areas that warrant further attention and remain underexplored. One of the main contributions of our study is the comprehensive examination and potential future directions of ovarian cancer immunotherapy. By analyzing complex immunogenic therapies for OC, we provide novel insights for reversing immune responses and identifying populations that may benefit from immunotherapy. Specifically, our study explores the combination of immune checkpoint inhibitors with other therapeutic approaches, such as vaccines, to enhance treatment efficacy. Furthermore, our study highlights potential biases and limitations present within existing bodies of literature. As we relied solely on Web of Science database and English language publications for research purposes, this could have caused us to miss important studies published in other languages or indexed in different databases.

## Conclusion

Using VOS Viewer 1.6.20 we explore trends and the status of immunotherapy research and ovarian cancer screening over the past five years, with a particular focus on contributions from the People’s Republic of China and Germany, both of which have been significant in this field. International cooperation played an essential role. As expected, the volume of research in this area continues to grow steadily over time. Prominent authors such as Zhang Y and Wang Y have emerged as frontrunners in this field, collectively contributing to an impressive 231 published documents during this period. Journal of Frontiers Immunology and Cancers in Switzerland Was among the top publications according to volume ranking. In terms of Citation rankings, the two most-cited articles ‘CAR-T cell therapy: current limitations and potential strategies; and Epithelial ovarian cancer: Evolution of management in the era of precision medicine.’ terms like ‘pan-cancer’, ‘prognostic signature’, and ‘necroptosis’ have emerged as significant indicators of hotspots in this field. These latest keywords highlight areas suitable for further investigation and guide researchers in determining priority research topics. In addition, identifying these hotspots is crucial for informing funding allocation decisions, ensuring that resources are directed toward the most promising and influential research areas. By focusing on these emerging trends and key areas, future research can strategically advance scientific progress in immunotherapy for OC and improve patient prognosis.

## Data Availability

The datasets presented in this study can be found in online repositories. The names of the repository/repositories and accession number(s) can be found below: https://webofscience.clarivate.cn/wos/woscc/summary/53fd6b71-1c72-46c4-9b85-ef630c7eb651-f5007bc3/relevance/1.
